# The Role of Ion-Transporting Proteins in Human Disease

**DOI:** 10.3390/ijms25031726

**Published:** 2024-01-31

**Authors:** Yoshinori Marunaka

**Affiliations:** 1 Medical Research Institute, Kyoto Industrial Health Association, 67 Kitatsuboi-cho, Nishinokyo, Nakagyo-ku, Kyoto 604-8472, Japan; marunaka@koto.kpu-m.ac.jp; 2 Research Organization of Science and Technology, Ritsumeikan University, Kusatsu 525-8577, Japan; 3 Graduate School of Medical Science, Kyoto Prefectural University of Medicine, Kyoto 602-8566, Japan

This Special Issue focuses on the significance of ion-transporting proteins, such as ion channels and transporters, providing evidence for their significant contribution to bodily and cellular functions via the regulation of signal transduction and ionic environments [1,2,3,4,5]. In the late 1980s, the cystic fibrosis transmembrane regulator (CFTR) gene and its most common cystic fibrosis (CF)-causing mutation (ΔF508) were discovered [6]. Subsequently, many other genes which encode proteins from ion-transporting channels and transporters have been found to be associated with human disease. Even though the discovery of genes which are associated with human diseases marks a major advance within the field, the development of therapies to treat such channelopathies and transportopathies has not yet been fully accomplished.

CF patients are well known for showing deficient water secretion in the lungs, the liver, the sinuses, the small and large intestines, pancreatic and hepatobiliary ducts and male reproductive tracts [7,8,9,10,11,12]. One of the most serious problems in patients with CF is death from pneumonia caused by impaired fluid secretion into the airway cavity, which leads to dry lungs and an increased susceptibility to infection [8]. A gene responsible for one of the most serious problems in CF patients, impaired fluid secretion into the airway cavity, has been cloned by a research group in Toronto, Canada [6], and the gene has been called the cystic fibrosis transmembrane regulator (CFTR) [6]. This discovery has clearly pointed out that CF is a genetic disease, and various types of CFTR mutations have been classified from a functional perspective [13,14,15,16]: e.g., G542, ΔF508, G551D, R117H, A445E [13] (Figure 1), 3849 + 10kbC > T and C1400X [14]. The most common CFTR mutation is ΔF508 (loss of phenylalanine at position 508), which leads to a deficiency in the amount of protein being trafficked to the cell membrane [14,15,16]. Thus, ΔF508 CFTR has no role in the secretion of Cl^−^ into the airway lumen [14,15,16]. Deficient Cl^−^ secretion into the airway lumen, resulting in impaired water secretion, leads to dryness in the airway lumen and causes bacterial and viral infections. Similar CFTR dysfunction is not only observed in the lung but is also present in other organs such as the liver, the sinuses, the intestine, the colon, hepatobiliary and pancreatic ducts and male reproductive tracts [8,9,10,11]. CFTR modulator therapy is currently implemented by enhancing CFTR function through two mechanisms [17]. Potentiators such as ivacaftor increase the probability of the channel being open, allowing Cl^−^ and HCO_3_^−^ to pass across the cell membrane and through the channel more easily. Correctors such as lumacaftor, tezacaftor and elexacaftor improve the number of CFTR Cl^−^ channels on the cell surface by helping proteins to fold properly, allowing the translocation of CFTR Cl^−^ channels to the cell membrane surface. Severe variants such as ΔF508 required both potentiators and correctors to ameliorate the quantity and function of channels at the membrane surface of cells. Currently, four modulators have been approved by drug regulatory agencies in Europe and the USA, and the indication for each therapeutic agent depends on the specific CFTR genetic variants present [18]. Further, Cl^−^ channels play a variety of important roles in bodily and cellular functions, such as regulation of cell volume [19,20] (Figure 2) and cell migration [21] (Figure 3).

Epithelial Na^+^ reabsorption in the renal collecting ducts contributes to blood pressure regulation by controlling the volume of bodily fluids [28]. Amiloride-sensitive epithelial Na^+^ channels in the renal epithelia contribute to Na^+^ reabsorption, and the mutation of amiloride epithelial Na^+^ channels causes disorders which affect blood pressure control. The amiloride-sensitive epithelial Na^+^ channel (ENaC) has been cloned by a research group in Lausanne, Switzerland, as it has been recognized as a causative gene for Liddle syndrome [29,30]. Each ENaC subunit consists of two transmembrane domains with intracellular N and C termini, and a large extracellular domain. The ion selectivity filter can specifically discriminate Na^+^, and the filter is located in the middle of the transmembrane domains [31]. The extracellular domain contains protease cleavage sites that enable its inhibitory effects on ENaC to be eliminated; it plays a key role in regulating ENaC activation [32,33]. The N-terminal ubiquitylation of the α and γ subunits has been implicated in the endocytosis and degradation of ENaC [34]; both the HGxxR sequence in the N-terminal and the PPPxY sequence in the C-terminal participate in regulating the ENaC. Mutations in the HGxxR sequence or the PPPxY sequence lead to the ENaC functioning abnormally, and are associated with the occurrence of Liddle syndrome [35] and pseudohypoaldosteronism (PHA) [36], i.e., the truncation of β or γ ENaC causes Liddle syndrome by elevating the amount of Na^+^ reabsorption in the kidney by increasing the number of functional ENaC located at the plasma membrane [36,37,38,39,40,41].

In addition to hypertension, ENaC plays important roles in human diseases [28] (Figure 4). In recent years, the ENaC has been found to contribute to immune cell activation, cystic fibrosis, endothelial cell dysfunction, pseudohypoaldosteronism (PHA), aggravated inflammation involved in high salt-induced hypertension, tumors and taste dysfunction [42]. ENaC hyperfunction elevates the concentration of intracellular Na^+^ ([Na^+^]_i_), leading to an intracellular-Ca^2+^ overload due to the activation of the Na^+^/Ca^2+^ exchanger; the overloaded intracellular Ca^2+^ is a key factor in ENaC-related inflammation [28].

The pH of interstitial fluid also plays an important role in various bodily and cellular functions [43,44,45,46,47]; lowering the pH of the interstitial fluid causes insulin resistance, and increases the accumulation of amyloid β, a candidate causative factor in Alzheimer’s Disease. The interstitial fluid’s pH is controlled by various types of ion transporters [48] (Figure 5), e.g., anion exchanger (AE), monocarboxylate transporter (MCT), Na^+^-HCO_3_^−^ cotransporter (NBC), Na^+^-driven Cl^−^/HCO_3_^−^ exchanger (NDCBE), Na^+^/H^+^ exchanger (NHE), H^+^-ATPase (H^+^ pump). Variations in the interstitial fluid’s pH are primarily affected by glucose metabolism performed via the anaerobic process mediated through the glycolytic pathway followed by the aerobic one through the Krebs (TCA) cycle [49,50,51]. The interstitial fluid’s pH-buffer capacity is very low compared with that of blood and/or the intracellular space [52,53,54,55,56,57,58,59,60] (Figure 5A). Therefore, the interstitial fluid’s pH changes more easily compared with the pH of blood and/or the intracellular space under metabolic disordered conditions [52,53,54,55,56,57,58,59,60] (Figure 5B). The pH of the fluid around enzymes and their substrates (proteins) can affect the binding affinity of enzymes to their substrates (proteins), influencing the activity of the enzyme by altering the protein tertiary structure [61]. For example, a lowered interstitial fluid pH diminishes the insulin’s binding affinity to its receptor, leading to insulin resistance [62] (Figure 5B).

The lowered interstitial fluid pH enhances the accumulation of amyloid-β which is observed in patients suffering with Alzheimer’s disease, and leads to hyperphosphorylation of the tau protein causing neural loss, neural inflammation and synaptic impairment, which are associated with behavioral abnormalities and cognitive decline [63,64,65,66,67,68,69,70]. Patients with type 2 diabetes are at high risk of developing Alzheimer’s disease [71,72,73,74,75,76]. Lowered interstitial fluid pH, an acidic condition, activates the β- and γ-secretases involved in the formation of amyloid-β from the amyloid precursor protein [77,78,79,80,81,82,83,84]. Thus, the acidic conditions which occur in type 2 diabetes patients increases the production of amyloid-β by activating the β- and γ-secretases [68,69,70,77]. Therefore, the interstitial fluid’s pH should be maintained within the normal range by various ion transporting proteins and elevating the pH-buffer capacity to ensure the maintenance of healthy body conditions [48,85,86] (Figure 5).

Thus, ion-transporting proteins’ dysfunction play important roles in human diseases. This Special Issue aims to provide insights into recent advances in the function and structure of ion-transporting proteins as they relate to human disease and the molecular mechanisms that cause ion-transporting-protein dysfunction. Finally, I would like to conclude my editorial by presenting the essence of the content of the articles in this Special Issue. Characteristics of the lysosomal cation channel TMEM175, a Parkinson’s disease-related protein and a promising drug target, is reported using a modified whole-cell patch clamp technique applied to lysosome [87]. The advantages of this new technique are described in this article [87]. NHE plays an important role in extrusion of H^+^ produced in the intracellular space [48] (Figure 5). Salari et al. [88] have provided a detailed report of the expression sites of NHE in the colon, indicating the significance of NHE expression. Connexins, unique hemichannels, are transmembrane proteins which form gap junctions in vertebrates, and allow cell–cell or/and paracrine communication by releasing ATP, glutamate and NAD^+^, thus regulating processes such as synaptic transmission and cell migration [89]. The Na^+^,K^+^-ATPase (Na^+^,K^+^-pump) maintains critical cellular functions by creating fundamental Na^+^ and K^+^ environments in the intracellular space. For example, the Na^+^,K^+^-ATPase creates ionic environments for the maintenance of voltage-dependent excitatory membrane function in nerve and muscle cells, the secondary active transport of Na^+^-coupled glucose and amino acids into epithelial cells and the intracellular concentration of Ca^2+^ in heart myocardia [90]. The review by Baloglu [90] focuses on the regulation of Na^+^,K^+^-ATPases in ischemic heart disease and discusses the regulation of Na^+^,K^+^-ATPases under conditions of myocardial stress and their therapeutic potential based on the perspective of hypoxia-inducible factors. A review about ENaC is also published in this Special Issue [91]. The significance of ENaC has been described above in the early sections of this editorial. This review [91] focuses on discussing some recent developments in the search for novel therapeutic agents. I hope the articles published in this Special Issue provide researchers with new insights into the roles of ion-transporting proteins in human diseases and their potential as therapeutics for human diseases.

## Figures and Tables

**Figure 1:**
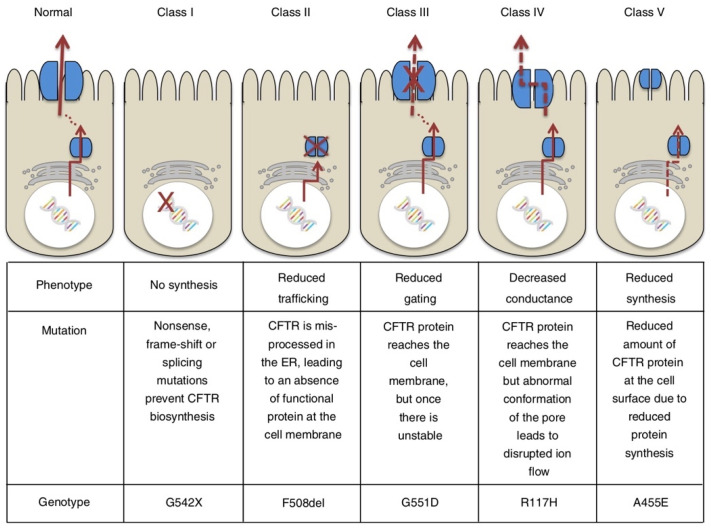
Classification of *CFTR* mutations. This figure has been published in an article by Koivula et al. [13], and is described under the terms of the Creative Commons Attribution 4.0 International License (http://creativecommons.org/licenses/by/4.0/) on 16 January 2024.

**Figure 2:**
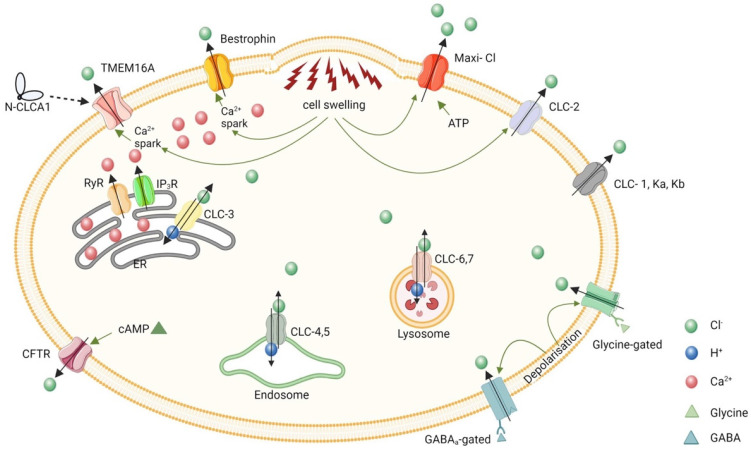
Six families of volume-regulated Cl^−^ channels. (i) TMEM16 family: calcium spark activates TMEM16A/Ano1, thereby leading to Cl^−^-efflux. (ii) Bestrophins: Bestrophins like Best1, are activated due to intracellular calcium spark or cell swelling and result in Cl^−^ efflux. (iii) Maxi-Cl: can be activated by ATP and/or cell swelling, resulting in the secretion of Cl^−^ in bulk amounts. (iv) CLC family: CLC-2 on the plasma membrane is activated by cell swelling, resulting in Cl^−^ efflux. CLC-3 in the ER is involved in the secretion of Cl^−^ in exchange for hydrogen ions, similarly to CLC-4 and 5 in the endosomes. CLC-6 and 7 are involved in the secretion of Cl^−^ from the lysosomes. (v) Ligand-gated Cl^−^ channels comprising glycine- and GABAa-gated Cl^−^ channels: when the ligands bind to the membrane receptor, the receptors act as Cl^−^ channels. (vi) CFTR: cytoplasmic cAMP activates the CFTR channel and thereby causes Cl^−^ efflux. ER: endoplasmic reticulum, RyR: ryanodine receptor, IP3R: Inositol-triphosphate receptor. Ion denotation: Red: Ca^2+^; Green: Cl^−^; Red: hydrogen ion; Blue: GABA ligand; Green: Glycine ligand. This figure has been published in an article by Sinha et al. [19], and is described under the terms of the Creative Commons Attribution 4.0 International License (http://creativecommons.org/licenses/by/4.0/) on 16 January 2024.

**Figure 3:**
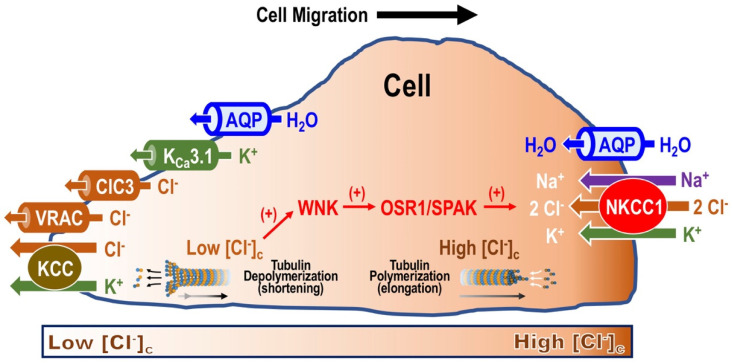
Changes to cell shape required during cell migration. Na^+^-K^+^-2Cl^−^ cotransporter 1 (NKCC1) and aquaporin (AQP) are expressed on the migrating-side membrane. NKCC1 is involved in Cl^−^ uptake into the cytosolic space with Na^+^ and K^+^ [22,23,24,25,26]. The uptake of these ions results in an influx of water into the cytosolic space via AQP due to an increase in cytosolic osmolarity [22]. The movement of Cl^−^, Na^+^, K^+^ and water causes an increase in cell volume accompanied by [Cl^−^]_c_ elevation, which promotes tubulin polymerization (elongation) [27] by inhibiting GTPase activity [21]. Like tubulin polymerization, actin monomers are enhanced to be polymerized. Then, cells migrate via these processes. On the one hand, K^+^-Cl^−^ cotransporter (KCC), volume-regulated anion channel (VRAC), Ca^2+^-activated K^+^ channel (K^+^_Ca_3.1) and AQP are expressed on the tail-end membrane during cell migration and Cl^−^ with K^+^ are excreted to the extracellular space via KCC, VRAC and K^+^_Ca_3.1 [22,26]. AQP-mediated water efflux to the extracellular space is caused by diminution in cytosolic osmolarity due to excretion of these ions. The movement of Cl^−^, K^+^ and water results in a decrease in cell volume accompanied by [Cl^−^]_c_ diminution, which leads to tubulin depolymerization (shortening) during the tail-end of the cell migration [27] by activating GTPase [21]. WNK activated by lowered [Cl^−^]_c_ induces phosphorylation (activation) of OSR1/SPACK, which increases NKCC1 activity by phosphorylating NKCC1 [23]. Thus, WNK is crucial within cell migration [23]. This figure has been published in an article by Marunaka [21] and is described under the terms of the Creative Commons Attribution 4.0 International License (http://creativecommons.org/licenses/by/4.0/) on 16 January 2024.

**Figure 4:**
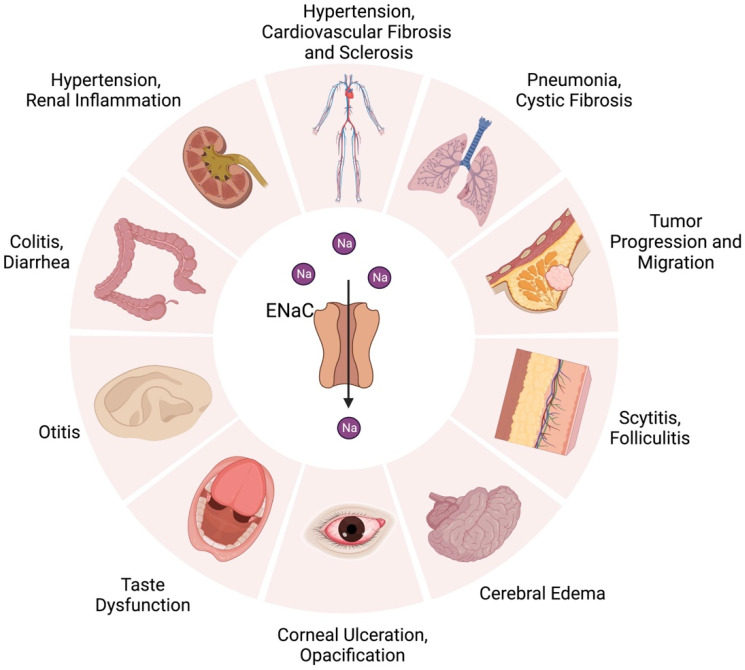
Distribution of ENaC in organs and related diseases. This figure has been published in an article by Chen et al. [28], and is described under the terms of the Creative Commons Attribution 4.0 International License (http://creativecommons.org/licenses/by/4.0/) on 16 January 2024.

**Figure 5:**
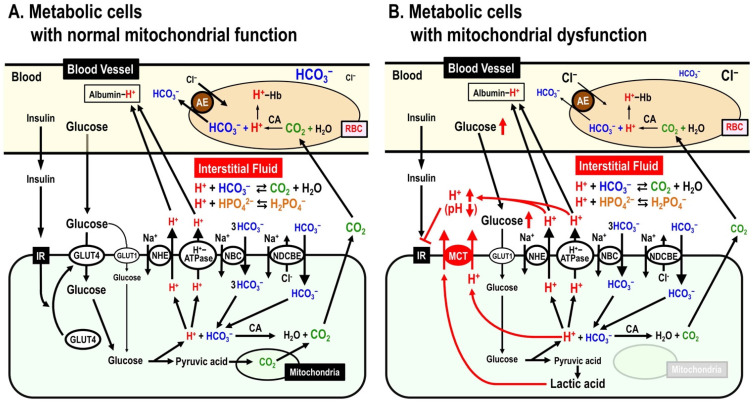
Mitochondrial dysfunction-induced insulin resistance via lowering interstitial fluid pH. (**A**) Metabolic cells with normal mitochondrial function. (**B**) Metabolic cells with mitochondrial dysfunction. AE, anion exchanger; CA, carbonic anhydrase; MCT, monocarboxylate transporter; NBC, Na^+^-HCO_3_^−^ cotransporter; NDCBE, Na^+^-driven Cl^−^/HCO_3_^−^ exchanger; NHE, Na^+^/H^+^ exchanger. This figure has been published in an article by Marunaka [48], and is described under the terms of the Creative Commons Attribution 4.0 International License (http://creativecommons.org/licenses/by/4.0/) on 16 January 2024.
